# Efficient Lossy Compression for Compressive Sensing Acquisition of Images in Compressive Sensing Imaging Systems

**DOI:** 10.3390/s141223398

**Published:** 2014-12-05

**Authors:** Xiangwei Li, Xuguang Lan, Meng Yang, Jianru Xue, Nanning Zheng

**Affiliations:** Institute of Artificial Intelligence and Robotics, Xi'an Jiaotong University, Xi'an 710049, China; E-Mails: xiangweili09@gmail.com (X.L.); jrxue@mail.xjtu.edu.cn (J.X.); nnzheng@mail.xjtu.edu.cn (N.Z.)

**Keywords:** compressive sensing imaging (CSI), lossy compression, CS acquisition, quantization, image processing

## Abstract

Compressive Sensing Imaging (CSI) is a new framework for image acquisition, which enables the simultaneous acquisition and compression of a scene. Since the characteristics of Compressive Sensing (CS) acquisition are very different from traditional image acquisition, the general image compression solution may not work well. In this paper, we propose an efficient lossy compression solution for CS acquisition of images by considering the distinctive features of the CSI. First, we design an adaptive compressive sensing acquisition method for images according to the sampling rate, which could achieve better CS reconstruction quality for the acquired image. Second, we develop a universal quantization for the obtained CS measurements from CS acquisition without knowing any *a priori* information about the captured image. Finally, we apply these two methods in the CSI system for efficient lossy compression of CS acquisition. Simulation results demonstrate that the proposed solution improves the rate-distortion performance by 0.4∼2 dB comparing with current state-of-the-art, while maintaining a low computational complexity.

## 1. Introduction

Digital image acquisition and processing is a traditional research topic and has been well studied in the past decades. A classical imaging system often contains two steps: acquiring amounts of raw image data in full spatial resolution by an image-sensor, and then massively dumpling the redundancy information of the raw image data in a compression process. According to the Shannon-Nyquist sampling theorem [1], the sampling rate of image acquisition needs to be at least twice as high as the highest frequency of the image signal so the image can be reconstructed accurately. The cost and computational complexity often rises greatly with the increase of camera resolution. Thus, it cannot meet well the requirements for many modern applications with energy and computational resource limitations, such as mobile terminal imaging [2], wireless multimedia sensor networks [3,4], space image acquisition [5], hyperspectral imaging [6,7], *etc.*

**Figure 1.**
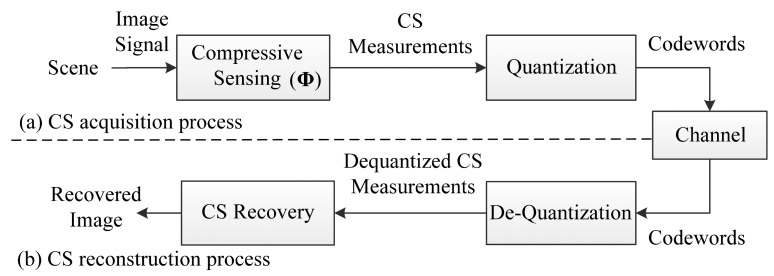
The framework of a typical CSI system. (**a**) CS acquisition process; (**b**) CS reconstruction process.

Compressive Sensing Imaging (CSI) is a new architecture for image acquisition and compression that has emerged in recent years, which enables acquiring and compressing a scene simultaneously [8–10]. Different from classical imaging solutions, CSI is able to acquire an image by measuring the scene a few times with a single-pixel camera [11] instead of sampling in high resolution with several million sensor elements, which breaks the traditional image acquisition architecture. With the lower sampling rate and fewer sensing elements in CSI [11], the imaging system is cheaper and less power is consumed. A typical CSI system mainly contains two processes, as shown in Figure 1. In the Compressive Sensing (CS) acquisition process, an input image is firstly measured via a measurement matrix **Φ** in a reduced dimensionality instead of full image resolution. Then the resulting CS measurements are quantized into a set of codewords, and these codewords are transmitted to the receiver via a channel. In CS reconstruction process, the received codewords are dequantized into CS measurements and the image is reconstructed by a CS recovery program.

Great effort has been put on the development of efficient CSI systems in recent years including the hardware application and algorithm design. A single-shot Complementary Metal-Oxide-Semiconductor (CMOS) image-sensor [12] performs CS at the Analog/Digital (A/D) conversion stage. Dadkhah *et al.* [13] reviewed different hardware implementations and important practical issues of CS encoding in CMOS sensor technologies. Chen *et al.* [14] solved the problem of wide-area video surveillance systems based on the parallel coded aperture CSI system. With all these CSI systems, the cost and complexity of image-sensor deployment could be well reduced and the low-complexity image/video acquisition can be designed by shifting the computational burden to the reconstruction process. An example is shown in [15] proving that CS provides great energy efficiency for sensing operations in Wireless Sensor Networks (WSNs).

In practical CSI applications, CS acquisition is assumed to be implemented in some analog image-acquisition hardware like a single-pixel camera [11]. The acquired CS measurements are real-valued, which has a large amount of data for storage and transmission. Therefore, the lossy compression of CS measurements is required in the CS acquisition process. The design of efficient lossy compression of CS acquisition will raise two questions: how to adaptively sparsify the image signal for better CS reconstruction, and how to efficiently quantize the real-valued CS measurements. We analyze these two questions in the following two paragraphs.

At the CS acquisition stage, it is known that a certain degree of sparsity of the original signal is important for CS reconstruction. If the original signal is not sparse enough, the reconstruction quality will degrade due to the *noise folding* effect. Arias-Castro *et al*. [16] studied this problem in a practical CS system. Laska *et al.* [17] showed that a compressible signal could only be recovered by part of its important coefficients, and the remaining coefficients will cause the *noise folding* effect, which seriously degrades the reconstruction quality. In order to reduce the *noise folding* effect, the simple Discrete Cosine Transform (DCT) coefficients truncation method [18] was applied in CS-based image/video coding to improve its Rate-Distortion (R-D) performance. However, it does not consider the variation of sampling rate which is the main factor for deciding how many important DCT coefficients can be accurately recovered for the reconstruction of the original signal. Mansour *et al*. proposed an adaptive compressive sensing method [19], which focuses on acquiring the large coefficients of a compressible signal to reduce the *noise folding* effect. However, this method simply used an empirical linear model to adapt the sampling rate.

At the CS measurements quantization stage, a general solution is to quantize the CS measurements with uniform scalar quantization considering the low-complexity requirements of the CSI system. However, it does not specifically consider the distribution characteristic of CS measurements. Goyal *et al.* [20] and Boufounos *et al.* [21] analyzed the quantization problem of CS measurements with a simple scalar quantization. The classical Probability Density Function (PDF)-based quantization [22] was adopted for this problem, which could exploit the distribution characteristic of the signal. Sun *et al.* [23] proposed an R-D optimized quantization for CS measurements based on the classical PDF-based quantization, which exploits the distribution characteristic of CS measurements. However, the direct implementation of PDF-based quantization will cause high computational complexity, since the PDF needs to be obtained in advance for CS measurements of each input image.

The main contribution of this paper is to develop an efficient lossy compression solution for CS acquisition of images in the CSI system, by considering both the image signal sparsification and CS measurements quantization. First, we propose an adaptive compressed sensing method to make the image signal sparser for CS acquisition, such that the reconstruction quality can be improved by reducing the *noise folding* effect. The proposed adaptive compressive sensing method truncates the image coefficients to retain some large coefficients according to the sampling rate. Second, we design a low-complexity universal quantization for the CS measurements by establishing a universal probability model without knowing any *a priori* information about the input image. Finally, the proposed adaptive compressive sensing and universal quantization methods are incorporated into the CSI system. Simulation results show that the proposed lossy compression solution for CS acquisition in the CSI improves its R-D performance and reduces its computational complexity compared with the conventional solution.

The rest of the paper is organized as follows: in Section 2, an adaptive compressive sensing method for CS image acquisition is presented. In Section 3, a universal quantization method is introduced for quantization of CS measurements. Then we provide the lossy compression solution of CS acquisition in the CSI system with the proposed adaptive compressive sensing and universal quantization methods in Section 4. Simulation results are shown in Section 5. Finally, we conclude the paper in Section 6.

## 2. Adaptive Compressive Sensing Method for CS Acquisition

In traditional signal acquisition systems, the analog signals are often low-pass filtered to limit their bandwidth before acquisition based on the Shannon-Nyquist sampling theorem [1]. The reconstruction quality could be improved by reducing the *aliasing* effect which is caused by the unlimited bandwidth of the signal. In a CS acquisition system, the reconstruction quality will degrade due to the *noise folding* effect caused by lack of sparsity of the signal. We will make the image sparser for CS acquisition in CSI system to improve the reconstruction quality. More specifically, we sparsify the image by retaining only a number of large coefficients according to the sampling rate.

### 2.1. Overview of Related Concepts in CS

The Compressive Sensing (also known as Compressive Sampling, CS) theory [24,25] enables to directly acquire the compressed signal with a few random projections and recover the signal from the projections. We suppose that **f** ∈ **ℜ***^N^* is a discrete signal, and denote its coefficients in the sparsifying basis **Ψ** ∈ **ℜ***^N^*^×^*^N^* by **x** ∈ **ℜ***^N^* Signal **f** is considered to be *k*-sparse with respect to **Ψ** if and only if *k* coefficients are non-zero. According to the CS theory, we can acquire the *k*-sparse signal **f** as follows:

(1) $$\begin{array}{ccc} \text{y}=\Phi \text{f} & \text{with} & y_{i}=\langle \phi_{i},\text{f}\rangle ,i=1\ldots n \end{array}$$

where **y** ∈ **ℜ***^n^* (*n* ≪ *N*) is the CS measurements and **Φ**=[*ϕ*_1_,*ϕ*_2_,…,*ϕ_n_*]*^T^* ∈ **ℜ***^n^*^×^*^N^* is the measurement matrix that is incoherent with **Ψ**. The Sampling Rate (SR) is defined as:

(2) SR=n/N

Supposing that **Φ** and **Ψ** satisfy the Restricted Isometry Property (RIP) condition of order *k* [24], then the coefficients **x** can be exactly recovered by solving the following optimization problem:

(3) $$\begin{array}{cccc} \underset{\text{x}\in {ℜ}^{N}}{\text{min}} & {∥\text{x}∥}_{1} & \text{s.t.} & \text{y}=\Phi \Psi \text{x} \end{array}$$

Finally the reconstructed signal is obtained as **f̃** = **Ψ**^–1^**x̃** with the solution **x̃** of Equation (3). In practical application, the coefficients **x** are not strictly sparse but compressible. In this case, the sorted coefficients of **x** in decreasing order often obey a power law [26]. Then **x̃** contains the most significant coefficients of **x**, which provides a good approximation of the signal [24,27]. Moreover, CS measurements **y** will be corrupted by quantization noise [28]. Thus, the practical CS acquisition model in Equation (1) can be described more precisely as:

(4) y=Φf+e

where **e** is the quantization noise bounded by a noise power ε.

Let *T_k_* be the indices of the largest *k* values of **x**, and **x***_T_k__* be the *k*-sparse approximation of **x**. Candès *et al.* [26] and Donoho [25] stated that if **Φ** and **Ψ** satisfy certain RIP condition and the number of CS measurements is sufficient enough, that is:

(5) n≥O(klog(N/k))

then *k* largest values of **x** can be recovered stably from *n* CS measurements by solving the relaxed *l*_1_ minimization problem [21]:

(6) $$\begin{array}{cccc} \underset{\text{x}\in {ℜ}^{N}}{\text{min}} & {∥\text{x}∥}_{1} & \text{s.t.} & {∥\Phi \Psi \text{x}-\text{y}∥}_{2}\leq ɛ \end{array}$$

The solution x_*_ to Equation (6) obeys

(7) $${∥{\text{x}}^{*}-\text{x}∥}_{2}\leq C_{0}ɛ+\frac{C_{1}}{\sqrt{k}}{∥{\text{x}}_{T_{k}^{c}}∥}_{1}$$

where *C*_0_ and *C*_1_ are constants.

Equation (7) shows that the reconstruction error of **x** depends on two error terms *C*_0_ε and $\frac{C_{1}}{\sqrt{k}}{∥{\text{x}}_{T_{k}^{c}}∥}_{1}$. The first term is proportional to the noise power and the second term is proportional to ${∥{\text{x}}_{T_{k}^{c}}∥}_{1}$ (the *l*_1_ norm of “tail” part of **x**), which will cause the *noise folding* effect [17]. The first error term will be considered in Section 3 to reduce the reconstruction error.

In this section, we consider the second term. Supposing the CS measurements **ỹ** = **ΦΨx***_T_k__* can be obtained from the *k*-sparse approximation **x***_T_k__* of **x**, we recover **x** with **ỹ** instead of **y**. The solution x^*^ to Equation (3) obeys ‖x^*^ – **x***_T_k__*‖_2_ = 0 and:

(8) $${∥{\text{x}}^{*}-\text{x}∥}_{2}={∥{\text{x}}_{T_{k}^{c}}∥}_{2}$$

**Figure 2.**
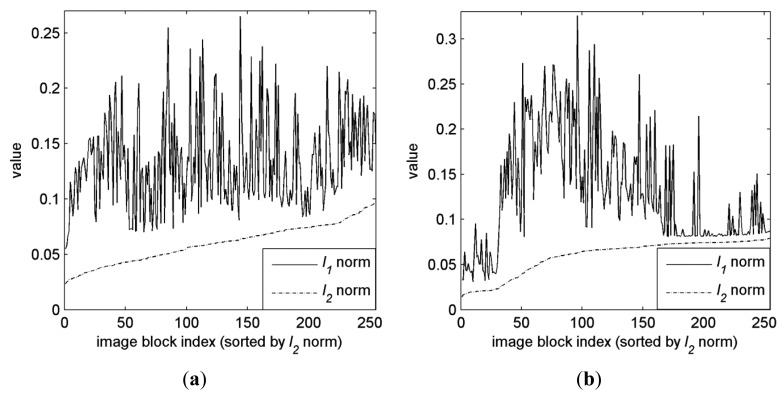
Comparison between *l*_1_ norm and *l*_2_ norm of DCT coefficients of 16 × 16 blocks in an image. (**a**) Lena (Figure 8a); (**b**) Cameraman (Figure 8b).

It is shown that solving Equation (3) with CS measurements **ỹ** results in an error in Equation (8). The error will be the second term in Equation (7) when **y** is used (without quantization). Generally, the *l*_1_ norm in Equation (7) is often greater than the *l*_2_ norm in Equation (8) for a “tail” part of the same compressible signal [19]. An example is shown in Figure 2, in which *l*_1_ norm is often greater than *l*_2_ norm for the DCT coefficients of 16 × 16 blocks in an image. So it is possible to achieve better CS reconstruction quality by recovering **x** with **ỹ** obtained from sparsified coefficients **x***_T_k__* In this section, we design an adaptive compressive sensing method which adaptively sparsifies the compressible signal for CS acquisition.

### 2.2. Adaptive Compressive Sensing Method

Generally, the conventional CS approach acquires all the values of the signal coefficients without sparsifying by truncating the small ones. If the signal is not sparse enough, it may result in poor reconstruction quality due to the *noise folding* effect, especially when the sampling rate is low. On the other hand, if we truncate too many values of the signal, the reconstruction quality may also degrade.

**Figure 3.**
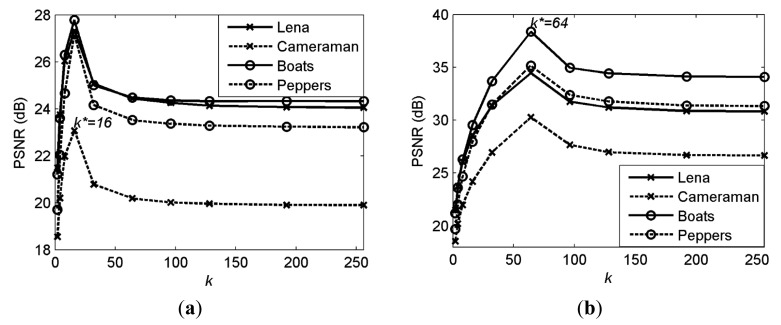
Reconstruction PSNR for different sampling rates on four test images. (**a**) *SR* = 1/4; (**b**) *SR* = 5/8.

**Figure 4.**
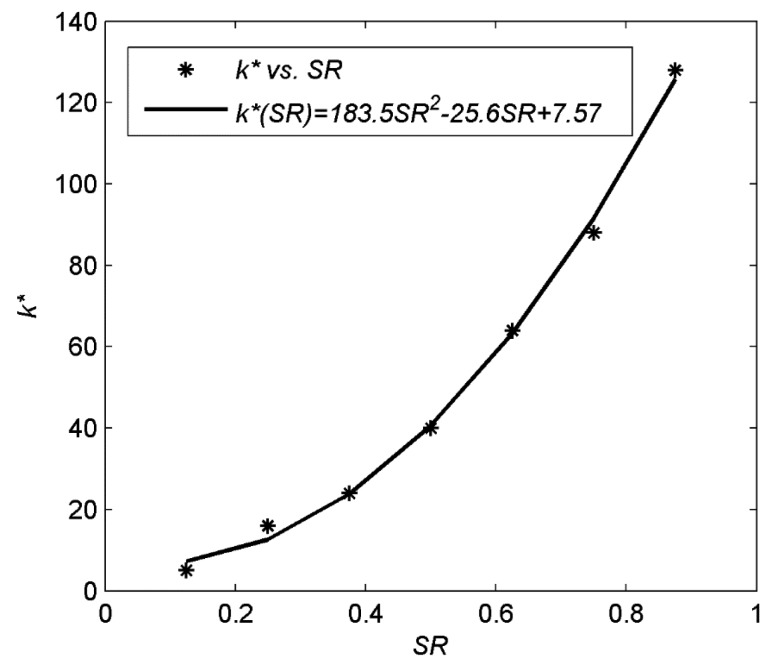
Empirical truncation point model.

We truncate a part of small values of the signal coefficients according to the sampling rate. To achieve this, we aim to find the optimal truncation point k^*^ as follows:

(9) $$k^{*}=\underset{k\in 1,2,\ldots ,N}{\text{arg min}}{∥{\tilde{\text{x}}}_{T_{k}}-\text{x}∥}_{2}$$

where **x̃***_T_k__* is the CS reconstruction of sparsified coefficients **x***_T_k__* via Equation (3) with CS measurements **ỹ** = **ΦΨx***_T_k__* ∈ **ℜ***^n^* and **Φ** ∈ **ℜ***^n^*^×^*^N^* The Sampling Rate (*SR*) is defined in Equation (2). Equation (9) can be solved by searching all the truncation points, which has a high computational complexity. Therefore, rather than solving Equation (9), we try to establish a truncation point model to describe the relationship between k^*^ and *SR* for images to reduce its computational complexity. We examine the performance of the CS reconstruction at different truncation points for the DCT coefficients of the image blocks. Different sampling rates are tested for each image. For each sampling rate, the optimal truncation point k^*^ is obtained from Equation (9). Figure 3 shows the reconstruction Peak Signal-to-Noise Ratio (PSNR) (PSNR is obtained by firstly calculating the mean squared error (MSE) between the reconstructed signal and the original signal and then transformed to PSNR). For *SR* = 1/4, 5/8 on four test images, in which the optimal truncation point is k^*^ = 16 for *SR* = 1/4 and k^*^ = 64 for *SR* = 5/8. We can observe that the optimal truncation point for different images mainly depends on the sampling rate. Therefore, we first calculate k^*^ (averaging the values for all test images) at different sampling rates, and then fit their relationship with a quadratic polynomial function as shown in Figure 4, in which the model has been established as k^*^(*SR*)=183.5·*SR*^2^–25.6·*SR*+7.57.

Once k^*^ is obtained, an optimal truncation indices T_k__*_ can be determined. Then **x** could be truncated with T_k__*_. Here, we define a truncating matrix as:

(10) $$\text{W}=\text{Diag}(\text{w})\in {ℜ}^{N\times N}$$

where:

(11) $$\begin{array}{cc} \text{w}={\left[w_{1},\ldots ,w_{i},\ldots ,w_{N}\right]}^{T}, & w_{i}=\{\begin{array}{cc} \begin{array}{cc} 1, & i\in T_{k^{*}}\\ 0, & i\in {T_{k^{*}}}^{c} \end{array}, & i=1\ldots N \end{array} \end{array}$$

Then the truncated coefficients can be calculated as:

(12) $${\text{x}}_{{\text{T}}_{k^{*}}}=\text{Wx}$$

The **x**_T_k___*_ in Equation (12) is the truncated coefficients from **x**. In the CS acquisition process, we can acquire **x**_T_k___*_ instead of **x** to reduce the reconstructed error. At the reconstruction stage, we recover the coefficients from the CS measurements **ỹ** = **ΦΨx**_T_k___*_, which could be solved as follows:

(13) $$\begin{array}{cccc} \underset{\text{x}\in {ℜ}^{N}}{\text{min}} & {∥\text{x}∥}_{1} & \text{s.t.} & \Phi \Psi \text{x}=ỹ \end{array}$$

The solution of Equation (13) is the reconstructed coefficients from the CS measurements **ỹ** = **ΦΨWx**

## 3. Proposed Universal Quantization for CS Measurements

In practical CSI system, the real-valued CS measurements **ỹ** = [*ỹ*_1_,…,*ỹ_i_*,…, *ỹ_n_*]*^T^* obtained in Section 2 need to be further quantized to codewords **ỹ***_Q_* = [*ỹ*_1,_*_Q_*,…,*ỹ_i,Q_*,…, *ỹ_n,Q_*]*^T^* for processing and transmission, which can be described as:

(14) $${ỹ}_{i,Q}=Q({ỹ}_{i}),i=1\ldots n$$

where *Q* is the quantization function. The efficient CS measurements quantization is an important part of the lossy compression for CS acquisition, which reduces the reconstruction error in Equation (7) as mentioned in Section 2. In this section, we proposed a universal quantization method for the CS measurements of any input image. For simplicity, we use *y_i_* and *y* instead of *ỹ_i_* and *ỹ_i_* in this section, respectively.

### 3.1. Universal Probability Modeling for CS Measurements

We first model the probability distribution of CS measurements, as it is related with the quantization design. Generally, we assume that the values of measurement matrix **Φ**=[*ϕ*_1_,…,*ϕ_i_*,…,*ϕ_n_*]*^T^*∈** ℜ ***^n^*^×^*^N^,i*=1…*n* are a Gaussian distribution with zero mean and variance 1/*n* [26,29]. Then it is easy to know that *y_i_* = *ϕ_i_***x**,*i* = 1…*n* is also a Gaussian distribution when the dimension *N* of the signal **x** ∈ **ℜ***^N^* is very large according to the Central Limit Theorem. That is *y_i_* ∼ N(0,σ^2^) [17], where the variance *σ*^2^ is related with CS measurements. Its Probability Density Function (PDF) is:

(15) $$f_{\text{N}(0,\sigma^{2})}(y)=\frac{1}{\sigma \sqrt{2\pi }}\exp\left(-\frac{y^{2}}{2\sigma^{2}}\right)$$

**Figure 5.**
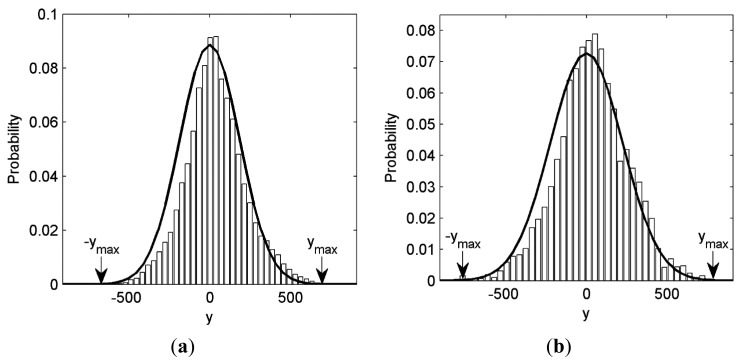
CS measurements histogram and the fitted curve. (**a**) Lena 256 × 256 (*SR* = 0.5); **(b)** Cameraman 256 × 256 (*SR* = 0.7).

The histogram of CS measurements from test images Lena and Cameraman are depicted in Figure 5, together with the fitted curve of *y_i_* ∼ N(0,σ^2^), where *σ*^2^ is the variance of CS measurements. It is shown that the CS measurement obeys Gaussian distribution well. In practical CSI system, the CS measurements are within the range [–*y*_max_, *y*_max_], where *y*_max_ is the maximal value of all CS measurements. The probability fraction that outside the range is often very small as shown in Figure 5. Thus we only consider the range [–*y*_max_, *y*_max_] in Equation (15). Although the real *y*_max_ is unknown in the modeling procedure, it can be approximated by *y*_max_ ≈ *d* · *σ* instead, where *d* is an empirical parameter ranging from 3 to 5 [30]. We firstly divide the range [–*d*·*σ,d*·*σ*] into *M*_max_ = 2^*R*_max_^ equal intervals, where *R*_max_>10 is a predefined large quantization rate (bits per CS measurement) for high-resolution approximation. Since the size Δ = *d*·*σ*/2^*R*_max_^^–1^ of the interval is small, the approximation error is bounded by Δ/2 Thus the probability *F_k_* that *y* will be contained in the *k*-th (*k* = 1,2,…,*M*_max_) interval can be approximated as follows:

(16) $$\begin{array}{ll} F_{k} & \approx f_{\text{N}(0,\sigma^{2})}(y_{\left(k\right)})\cdot \Delta \\ & =\frac{d}{2^{R_{\max}-1}}\cdot \frac{1}{\sqrt{2\pi }}\exp\left(-\frac{{y_{\left(k\right)}}^{2}}{2\sigma^{2}}\right) \end{array}$$

where *y*_(_*_k_*_)_ ∼ N(0,*σ*^2^) is the center point of *k*-th interval.

Let *y′*_(_*_k_*_)_ = *y*_(_*_k_*_)_/*σ*, then we know that *y′*_(_*_k_*_)_ ∼ N(0,1). Then Equation (16) can be rewritten as:

(17) $$\begin{array}{cc} F_{k}=\frac{d}{2^{R_{\max}-1}}\cdot f_{\text{N}(0,1)}(y{'}_{\left(k\right)}), & k=1,2\ldots ,M_{\max} \end{array}$$

where *f*_N(0,1)_(y*′*_(_*_k_*_)_) is the standard Gaussian distribution.

It is shown in Equation (17) that *F_k_* has no relationship with the acquired CS measurements or image. It can be immediately calculated from the standard Gaussian distribution. Then the discrete probability model of the CS measurements can be obtained in advance without knowing any information about the input image. This benefits the low-complexity quantization design.

### 3.2. Universal Quantization Design

Based on the universal probability model *F_k_* Equation (17) derived above, we then implement the traditional PDF-based quantization [22] to optimize the R-D performance. However, this optimization problem has a higher computational complexity than other simple solutions, such as uniform quantization, *etc.* We aim to design an efficient look-up table based on the PDF-based quantization, such that the practical quantization of CS measurement can be achieved by a simple mapping operation.

From quantization theory [22], the PDF-optimized quantization can be obtained by solving the following optimization problem:

(18) $$\underset{Q}{\min}\left\|y-Q(y)\right\|$$

The solution of Equation (18) is as follows [22]:

(19) $$\lambda (y)=\frac{{f_{\text{y}}}^{1/3}(y)}{\int {f_{\text{y}}}^{1/3}(y')dy'}$$

where *f*_y_(*y*) is the PDF of the source *y*, and *λ*(*y*) is the function to determine the number of the quantization levels. Integrating *λ*(*y*) gives the fraction of the quantization reproduction levels. We equally divide the range *y*∈[–*d*·*σ,d*·*σ*] into *M*_max_ = 2^R_max_^ intervals Δ = *d*·*σ*/2^*R*_max_^^–1^. Denoting *y*_(_*_k_*_)_ to be the center point of *k*-th (*k*=1,2,…,*M*_max_) interval, then the fraction *λ_k_* of the quantization reproduction levels in the *k*-th interval can be approximated as follows:

(20) $$\begin{array}{ll} \lambda_{k} & \approx \lambda (y_{\left(k\right)})\cdot \Delta \\ & =\frac{{\left[f_{\text{N}(0,\sigma^{2})}(y_{\left(k\right)})\cdot \Delta \right]}^{1/3}}{\sum_{<M_{\max}>}{\left[f_{\text{N}(0,\sigma^{2})}(y_{\left(k\right)})\cdot \Delta \right]}^{1/3}} \end{array}$$

Substituting Equation (20) with Equation (17), we can derive:

(21) $$\begin{array}{cc} \lambda_{k}\approx \frac{{F_{k}}^{1/3}}{\sum_{<M_{\max}>}{F_{k}}^{1/3}}, & k=1,2\ldots ,M_{\max} \end{array}$$

Then the cumulative fraction Γ*_K_* of the quantization reproduction levels from the first interval to the *k*-th interval can be calculated as follows:

(22) $$\begin{array}{cc} \Gamma_{K}=\sum_{k=1}^{k=K}\lambda_{k}, & K=1,2\ldots ,M_{\max} \end{array}$$

**Figure 6.**
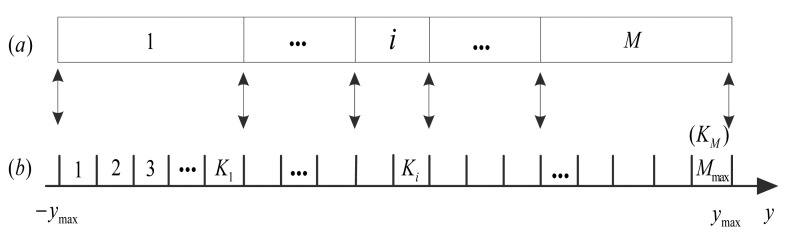
Quantization mapping table for the given target bits *R*. (**a**) Quantization cells; (**b**) Quantization intervals.

Since Γ*_K_* is only related to universal probability model *F_k_* which can be calculated by Equation (17) in advance, the designed quantization can be implemented efficiently as follows. For a given target quantization bits *R* (1<*R*≤*R*_max_), the *M* = 2*^R^* quantization cells can be mapped from the *M*_max_ = 2^*R*_max_^ intervals via a mapping table, as shown in Figure 6. The interval index *K_i_* in Figure 6b, which represents the rightmost interval for the *i*-th (1≤*i*≤*M*) quantization cell in Figure 6a is obtained as follows:

(23) $$K_{i}=\underset{K\in 1,2,\ldots ,M}{\arg\min}\left(\Gamma_{K}-\frac{i}{M}\right)$$

Note that the interval index *K_i_* (1≤*i*≤*M*) of *R* bits quantization is universal for the CS measurements of any input images. When quantizing a CS measurement *y* with the real value of *y*_max_ from specific image at the encoder, it is firstly quantized to an interval *K_i_* in Figure 6b, and then it is mapped to the *i*-th quantization cell in Figure 6a. Then the output codeword *i* is transmitted to the decoder via a channel. At the decoder, the quantization reproduction value *ŷ* of the received quantization cell *i* can be reconstructed as follows:

(24) $$\begin{array}{cc} {ŷ}_{\left(i\right)}=(K_{i-1}+K_{i})\cdot \Delta /2-y_{\max}, & i=1,\ldots ,M \end{array}$$

It is known that, the universal probability model *F_k_* and *λ_k_* in Equations (17) and (21) can be calculated in advance without knowing any information about the input image. Thus the cumulative fraction Γ*_K_* can be calculated in advance. Then the designed quantization is only related with the maximum value of the CS measurements *y*_max_ and so is the de-quantization in Equation (24). Therefore, for quantizing the real-valued CS measurements from an input image, *y*_max_ is firstly obtained, then the quantization cell with given target bits *R* can be obtained easily via a linear mapping operation. The computational complexity of this quantization method is low, compared with classical PDF-optimized quantization, which has to first estimate the PDF and then calculate the quantization function in Equation (18) for each input image.

## 4. Lossy Compression Solution for CS Acquisition with the Proposed Methods

**Figure 7.**
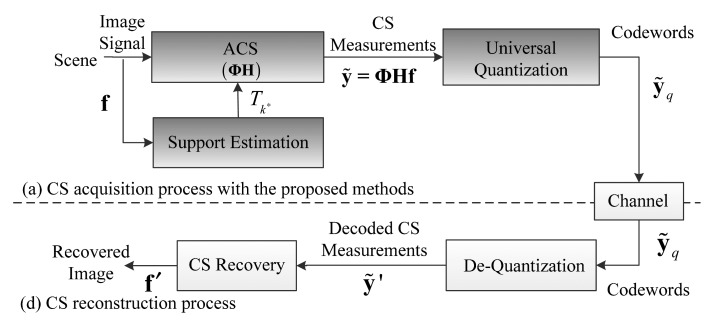
The framework of the CSI system with the proposed methods. (**a**) CS acquisition process with the proposed methods; (**b**) CS reconstruction process.

We incorporate the proposed Adaptive Compressive Sensing (ACS) and universal quantization methods into the CS acquisition process of CSI system to verify its lossy compression performance, as shown in Figure 7.

We design the ACS module for CS acquisition, which adaptively sparsifies the input image by truncating image coefficients in Figure 7a. In order to truncate the coefficients, ACS module requires optimal truncation indices T_k*_ to form a truncating parameter in Equation (10), so we design a support estimation module to estimate T_k*_ with truncation point model before acquisition in Figure 7a. After ACS module gets the estimated T_k*_ from support estimation module, the coefficients of the input image is then truncated and acquired. The resulting CS measurements are quantized by the proposed universal quantization module.

The procedure of this CSI system can be described as follows: denoting the image as **f**, the optimal truncation indices T_k*_ of **f** is first estimated by support estimation module. We build the truncating matrix **W** according to Equations (10) and (11) with T_k*_ and generate a matrix **H** = **Ψ^T^WΨ** in ACS module to truncate the coefficients of the image. The CS measurements **ỹ** = **ΦHf** are quantized into codewords **ỹ***_q_* by universal quantization module. At the decoder, **ỹ***_q_* are de-quantized to **ỹ′** by de-quantization module and finally **f′** is reconstructed.

In practical application, we further consider the following two aspects to reduce the computational complexity. Firstly, we calculate the truncation point model in Equation (9) in advance for images which is stored and used by support estimation module. Secondly, the optimal truncation indices T_k*_ of the coefficients can be approximately obtained from the partially acquired CS measurements at the sampling rate *SR* = 0.1 with [31]. In this paper, we use the first *k* lowest frequency indices of DCT coefficients of the image in the zig-zag order.

## 5. Simulation Results and Analysis

**Figure 8.**
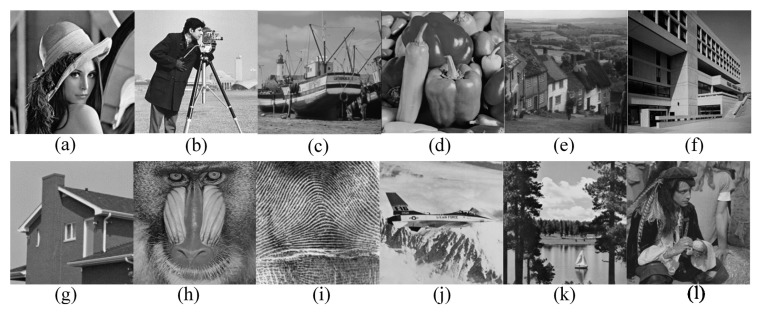
Gray scale test images. (**a**) Lena; (**b**) Cameraman; (**c**) Boats. (**d**) Peppers; (**e**) Goldhill; (**f**) Bank; (**g**) House; (**h**) Baboon; (**i**) Fingerprint; (**j**) Jetplane; (**k**) Lake; (**l**) Pirate.

We verify the performance of the proposed solution for CS acquisition in CSI system in the following three aspects: (a) the proposed ACS method; (b) the proposed universal quantization method; (c) the R–D performance of the lossy compression for CS acquisition with proposed ACS and universal quantization methods. Twelve grayscale images of 256 × 256 resolution with different spatial characteristics are used for simulation as shown in Figure 8.

In the simulation, the images are first normalized to unit *l*_2_ norm and then divided into 16 × 16 blocks. The values of the measurement matrix **Φ** are Gaussian distributed with zero mean and unit variance. We choose Discrete Cosine Transform (DCT) matrix as sparsifying basis **Ψ.** The CS scheme is performed over all blocks using the same measurement matrix **Φ** [32]. The standard *l*_1_ minimization program [33] is used as the CS recovery algorithm. We measure the reconstruction performance in terms of a Peak Noise-to-Signal Ratio (PSNR) between the reconstructed and original images. All simulations were implemented using MATLAB R2011b and carried out on a computer with dual core CPU at 2.4 GHz and 2 GB RAM.

### 5.1. Performance of Proposed ACS Method

We compare the proposed ACS method (denoted “Proposed”) with two methods: (a) traditional CS method without adaptive technique (denoted “Baseline”); (b) the solution in [19] (denoted “Method [19]”). The simulation results are shown in Table 1. Our method achieves an average of 3.21∼3.63 dB PSNR gain comparing to other solutions at *SR* = 1/4, and 3.37∼3.87 dB PSNR gain at *SR* = 5/8.

**Figure 9.**
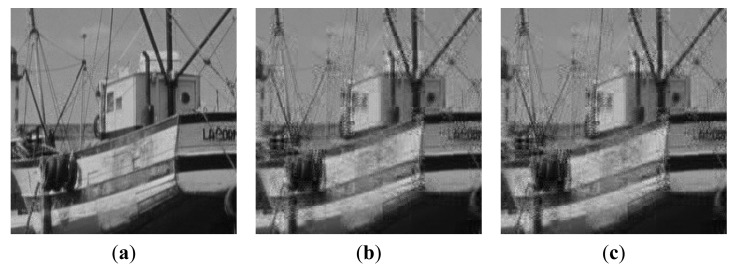
Subjective quality comparison on image Boats at *SR* = 1/4. (**a**) Proposed; (**b**) Method [19]; (**c**) Baseline.

**Figure 10.**
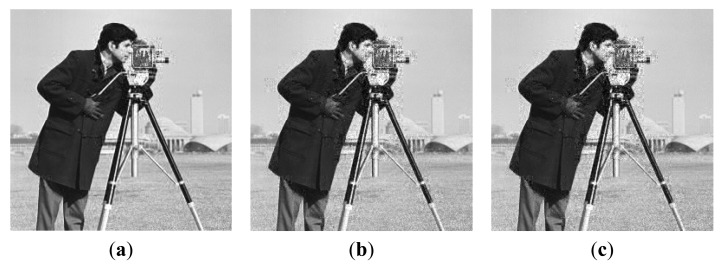
Subjective quality comparison on image Cameraman at *SR* = 5/8. (**a**) Proposed; (**b**) Method [19]; (**c**) Baseline.

Figures 9 and 10 show the subjective quality of the reconstructed images for Boats and Cameraman at *SR* = 1/4 and *SR* = 5/8. We can see that our method provides superior visual quality of recovered images compared to other solutions.

### 5.2. Performance of Proposed Universal Quantization Method

**Figure 11.**
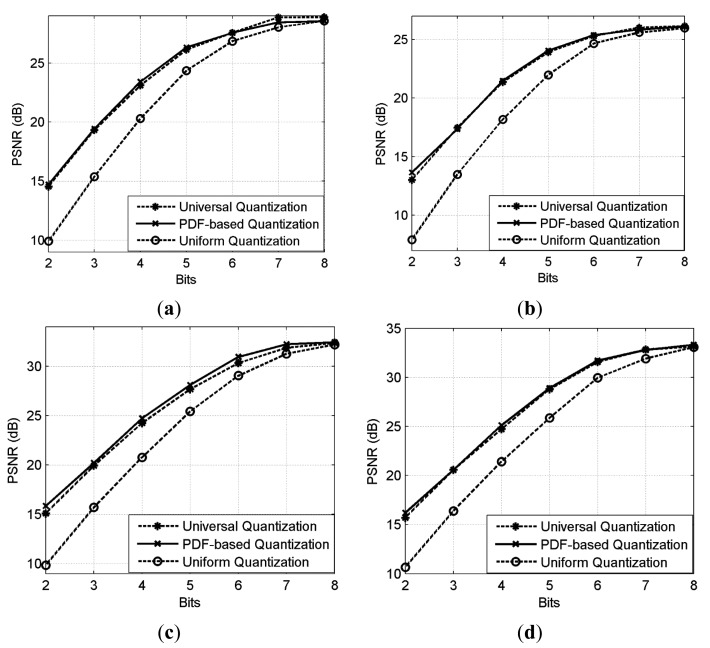
R-D performance for three methods after CS reconstruction. (*SR* = 0.7). (**a**) Lena; (**b**) Cameraman; (**c**) Boats; (**d**) Peppers.

**Figure 12.**
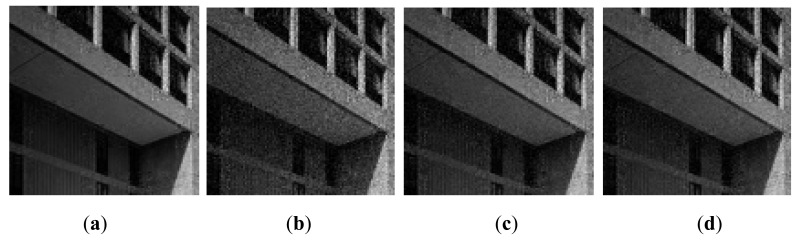
Subjective comparison (PSNR) of portions of Bank (*SR* = 0.7, *R* = 5) after CS reconstruction. (**a**) no quantization (26.73 dB); (**b**) uniform quantization (23.34 dB); (**c**) PDF-based quantization (24.91 dB); (**d**) universal quantization (24.90 dB).

**Figure 13.**
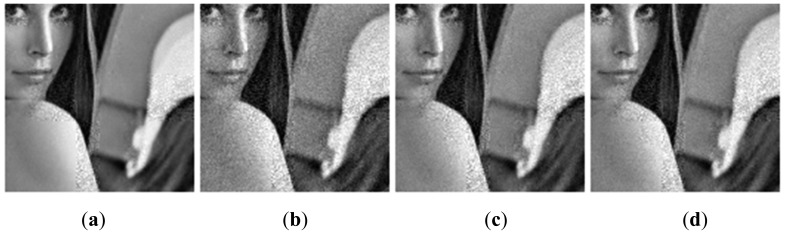
Subjective comparison (PSNR) of portions of Lena (*SR* = 0.7, *R* = 5) after CS reconstruction. (**a**) no quantization (27.92 dB); (**b**) uniform quantization (24.34 dB); (**c**) PDF-based quantization (26.32 dB); (**d**) universal quantization (26.13 dB).

The proposed universal quantization is compared to the uniform quantization and classical PDF-based quantization at fixed rate (without entropy coding). We empirically set *d* = 4.5 in Equation (17) for simulation. The quantization bits *R* is ranging from 2 to 8. The results are shown in Table 2 and Figure 11. Figures 12 and 13 show the subjective comparisons of these three methods. It is shown that the performance of our method is comparable with that of PDF-based quantization. Note that, the probability model in our method is established in advance without knowing any information about input image, while the probability model in PDF-based quantization needs to be calculated for each input image.

We further verify the computational complexity of the proposed universal quantization. Our method only requires a simple look-up table operation for CS measurements compression. In contrast, PDF-based quantization has to estimate the PDF for the CS measurements of each input image first and then calculate the quantization. Both of these two procedures have high computational complexity. The simulation times (with MATLAB) and theoretical complexity of universal quantization, uniform quantization, and PDF-based quantization are listed in Table 3. It is shown that the simulation time of the universal quantization is much lower than that of PDF-based quantization. Moreover, the computational complexity of the PDF-based quantization increases rapidly with the increase of the image resolution. By comparison, the computational complexity of the proposed universal quantization has no relation with the resolution of the input image. Comparing with uniform quantization, the simulation time of the universal quantization is a little high due to the look-up table operation; however, the R-D performance of the universal quantization is much better.

### 5.3. R-D performance of the Lossy Compression Solution of CS Acquisition

We evaluate the R-D performance of the proposed lossy compression solution of CS acquisition in CSI, which incorporates both the proposed ACS and universal quantization methods. We first examine the performance of the proposed CSI system in Figure 7 (denoted “Proposed”) and compare it with that of the traditional CSI system in Figure 1 (denoted “Baseline”), which uses the uniform quantization. The CS measurements are directly quantized without entropy coding. The sampling rate is *SR* =1/4, 3/8 and quantization bits *R* = 4, 5. Table 4 shows the results for eleven images. It is shown that the proposed solution increases the PSNR by 1.4 dB on average comparing to Baseline for *SR* = 3/8 and *R* = 5, and 1.0 dB for *SR* = 1/4 and *R* = 4. Moreover, we found that the PSNR gain for Bank is higher than that for Baboon, possibly because there are more texture details in Baboon (corresponding to more high frequency components), which is truncated by the ACS method.

We then verify the performance of the proposed solution in practical compression application by incorporating Differential Pulse Code Modulation (DPCM) [34,35] into the proposed and baseline CSI systems for compression (denoted by “Proposed CSI + DPCM” and “Baseline CSI + DPCM” respectively). In “Proposed CSI + DPCM”, the real-valued CS measurements are compressed by DPCM encoder, and the reconstructed image is obtained by CS recovery.

**Figure 14.**
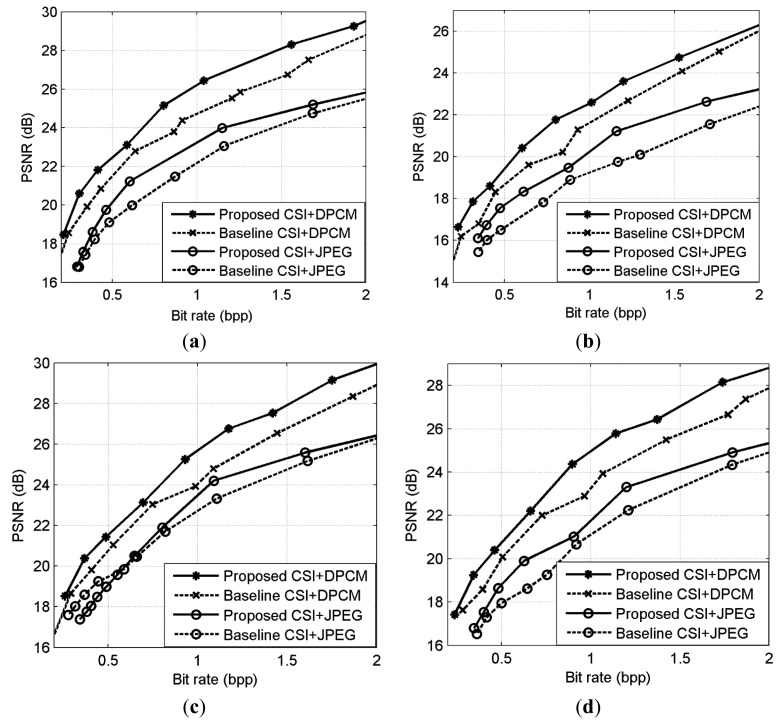
R-D performance comparison. (**a**) Lena; (**b**) Cameraman; (**c**) Boats; (**d**) Peppers.

**Figure 15.**
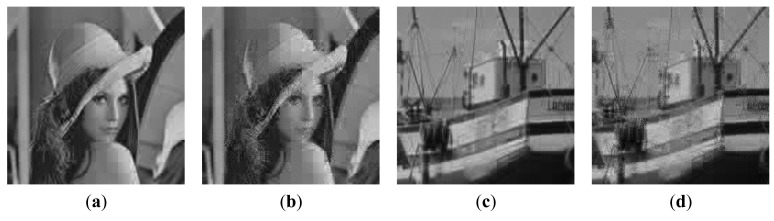
Reconstructed image with *SR* = 1/4 and *R* = 6. Lena: (**a**) Proposed CSI + DPCM (PSNR = 26.44 dB); (**b**) Baseline CSI + DPCM (PSNR = 23.78 dB); Boats: (**c**) Proposed CSI + DPCM (PSNR = 26.76 dB); (**d**) Baseline CSI + DPCM (PSNR = 23.92 dB).

**Figure 16.**
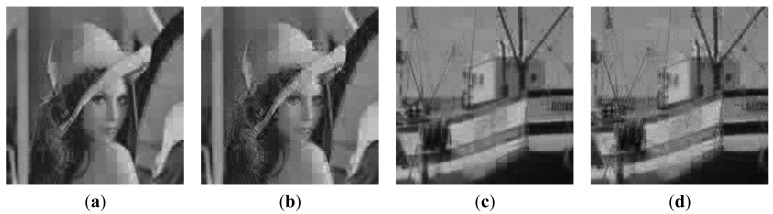
Reconstructed image at *SR* = 1/4 and JPEG quality level = 1/10. Lena: (**a**) Proposed CSI + JPEG (PSNR = 23.99 dB); (**b**) Baseline CSI + JPEG (PSNR=23.05 dB); Boats: (**c**) Proposed CSI + JPEG (PSNR = 24.20 dB); (**d**) Baseline CSI + JPEG (PSNR = 23.32 dB).

The R-D curve is the combination of all the best R-D points for each bit rate by searching all the sampling rates *SR* = {1/8,2/8,3/8,4/8,5/8,6/8,7/8} and quantization bits *R* = {1,2,3,4,5,6,7,8,9} [36]. Figure 14 shows the results for 4 images. We can see that the “Proposed CSI + DPCM” achieves up to 2dB PSNR gain comparing to “Baseline CSI + DPCM” solution. We further verify the performance of the proposed solution by incorporating JPEG [37,38] into the proposed and baseline CSI systems for compression (denoted by “Proposed CSI + JPEG” and “Baseline CSI + JPEG” respectively). The range of real-valued CS measurements is first mapped into 8-bit range 0–255, and then the CS measurements are sent to JPEG. The result is shown in Figure 14. It is shown that the “Proposed CSI + JPEG” achieves up to 2dB PSNR gain comparing to the “Baseline CSI + JPEG”. We also found that the“Proposed CSI + DPCM” outperforms “Proposed CSI + JPEG”. It is possibly because JPEG is designed for natural image compression, whose distribution characteristic is different from that of the CS measurements. The subjective results are shown in Figures 15 and 16, in which the proposed solution achieves better visual quality than that of the baseline solution.

## 6. Conclusions

In this paper, we have developed an ACS method and a universal quantization method for efficient lossy compression of CS acquisition in CSI systems. Simulation results show that the proposed solution achieves improved R-D performance and subjective quality of the CSI system; meanwhile, it has a low computational complexity.

## Figures and Tables

**Table 1.** PSNR (dB) comparison between the proposed method, baseline and Method [19].

**Sampling Rate**	**Methods**	**Lena**	**Cameraman**	**Boats**	**Peppers**	**Average**
*SR* = 1/4	Baseline	24.05	19.90	24.32	23.22	22.87
	Method [19]	24.49	20.33	24.63	23.71	23.29
	Proposed	27.64	23.17	28.29	26.91	26.50

*SR* = 5/8	Baseline	30.84	26.66	34.10	31.33	30.73
	Method [19]	31.34	27.14	24.54	31.91	31.23
	Proposed	34.50	30.29	38.42	35.18	34.60

**Table 2.** The PSNR (dB) gains over “uniform quantization” at *SR* = 0.7.

**Images**	***R*** ** = 3**		***R*** ** = 5**	
	
	**PDF-Based Quantization**	**Universal Quantization**	**PDF-Based Quantization**	**Universal Quantization**
Lena	4.06	3.95	1.98	1.79
Cameraman	3.87	3.97	1.57	1.56
Boats	4.51	4.23	2.68	2.25
Pepper	4.20	4.18	3.02	2.93
Goldhill	5.58	4.89	3.62	3.22
Bank	4.91	4.00	2.59	1.57
House	5.32	4.63	3.36	3.56
Baboon	5.28	4.48	1.84	1.87
Fingerprint	4.74	4.06	2.22	2.07
Jetplane	6.63	5.07	3.63	3.55
Lake	3.22	4.08	1.77	1.61
Pirate	6.31	5.24	4.46	3.42
Average	4.89	4.40	2.70	2.45

**Table 3.** Complexity comparison of three methods (*R* = 5, *SR* = 0.5).

**Methods**	**Theoretical Complexity**	**Simulation Time (ms)**		

		**Baboon 128 × 128**	**Bank 256 × 256**	**Cameraman 512 × 512**
PDF-based Quantization	*O*(*n*)	1314	4517	20174
Uniform Quantization	*O*(1)	0.02	0.02	0.02
Universal Quantization	*O*(1)	2.84	2.89	2.88

**Table 4.** PSNR (dB) comparison of the proposed solution and baseline solution.

**Images**	***SR*** ** = 3/8,** ***R*** ** = 5**			***SR*** ** = 1/4,** ***R*** ** = 4**		
	
	**Baseline**	**Proposed**	**Gain**	**Baseline**	**Proposed**	**Gain**
Lena	24.1	25.6	+1.5	20.4	21.7	+1.3
Cameraman	21.2	22.6	+1.3	18.1	18.7	+0.6
Boats	24.6	26.0	+1.4	20.8	21.6	+0.8
Peppers	23.8	25.3	+1.5	19.7	20.9	+1.2
Goldhill	25.1	26.5	+1.4	21.1	22.4	+1.3
Bank	21.9	23.7	+1.8	18.4	20.0	+1.6
House	26.3	27.6	+1.3	21.6	22.9	+1.3
Baboon	24.0	24.9	+0.9	21.3	21.7	+0.4
Fingerprint	20.9	22.3	+1.4	17.8	18.8	+1.0
Lake	22.9	24.4	+1.5	19.1	20.2	+1.1
Pirate	24.6	26.1	+1.5	20.6	22.0	+1.4
Average	23.6	25.0	+1.4	20.0	21.0	+1.0

## References

[1] Unser M. (2000). Sampling-50 years after Shannon. Proc. IEEE.

[2] Li C., Jiang H., Wilford P., Zhang Y., Scheutzow M. (2013). A new compressive video sensing framework for mobile broadcast. IEEE Trans. Broadcast..

[3] Akyildiz I.F., Melodia T., Chowdhury K.R. (2007). A survey on wireless multimedia sensor networks. Comput. Netw..

[4] Pudlewski S., Melodia T., Prasanna A. (2012). Compressed-sensing enabled video streaming for wireless multimedia sensor networks. IEEE Trans. Mob. Comput..

[5] Blanes I., Magli E., Serra-Sagrista J. (2014). A tutorial on image compression for optical space imaging systems. IEEE Trans. Geosci. Remote Sens. Mag..

[6] Klein M.E., Aalderink B.J., Padoan R., de Bruin G., Steemers T.A. (2008). Quantitative Hyperspectral Reflectance Imaging. Sensors.

[7] Willett R., Duarte M., Davenport M., Baraniuk R. (2014). Sparsity and structure in hyperspectral imaging. IEEE Signal Process. Mag..

[8] Wakin M., Laska J., Duarte M., Baron D., Sarvotham S., Takhar D., Kelly K., Baraniuk R. An architecture for compressive imaging.

[9] Romberg J. (2008). Imaging via compressive sampling. IEEE Signal Process. Mag..

[10] Takhar D., Laska J.N., Wakin M.B., Duarte M.F., Baron D., Sarvotham S., Kelly K.F., Baraniuk R.G. (2006). A new compressive imaging camera architecture using optical-domain compression. Proc. IS&T/SPIE Symp. Electron. Imag..

[11] Duarte M., Davenport M., Takhar D., Laska J., Sun T., Kelly K., Baraniuk R. (2008). Single-pixel Imaging via Compressive Sampling. IEEE Signal Process. Mag..

[12] Oike Y., Gamal A. (2013). CMOS image sensor with per-column ΔΣ ADC and programmable compressed sensing. IEEE J. Solid-St. Circ..

[13] Dadkhah M., Deen M., Shirani S. (2013). Compressive sensing image sensors-hardware implementation. Sensors.

[14] Chen J., Wang Y., Wu H. (2012). A coded aperture compressive imaging array and its visual detection and tracking algorithms for surveillance systems. Sensors.

[15] Razzaque M., Dobson S. (2014). Energy-efficient sensing in wireless sensor networks using compressed sensing. Sensors.

[16] Arias-Castro E., Eldar Y. (2011). Noise folding in compressed sensing. IEEE Signal Process. Lett..

[17] Laska J., Baraniuk R. (2012). Regime change: Bit-depth *versus* measurement-rate in compressive sensing. IEEE Trans. Signal Process..

[18] Zhang Y., Mei S., Chen Q., Chen Z. A novel image/video coding method based on Compressed Sensing theory.

[19] Mansour H., Yilmaz O. Adaptive compressed sensing for video acquisition.

[20] Goyal V., Fletcher A., Rangan S. (2008). Compressive sampling and lossy compression. IEEE Signal Process. Mag..

[21] Boufounos P.T., Baraniuk R.G. Quantization of Sparse Representations.

[22] Gray R., Neuhoff D. (1998). Quantization. IEEE Tran. Int. Theory.

[23] Sun J., Goyal V. Optimal Quantization of Random Measurements in Compressed Sensing.

[24] Candès E.J., Romberg J., Tao T. (2006). Robust uncertainty principles: Exact signal reconstruction from highly incomplete frequency information. IEEE Tran. Int. Theory.

[25] Donoho D.L. (2006). Compressed sensing. IEEE Tran. Int. Theory.

[26] Candès E.J., Romberg J., Tao T. (2006). Stable signal recovery from incomplete and inaccurate measurements. Comm. Pure Appl. Math..

[27] Cohen A., Dahmen W., DeVore R. (2009). Compressed sensing and best k-term approximation. J. Am. Math. Soc..

[28] Candès E., Tao T. (2005). Decoding by linear programming. IEEE Trans. Inf. Theory.

[29] Li X., Lan X., Yang M., Xue J., Zheng N. Universal and low-complexity quantization design for compressive sensing image coding.

[30] Rees D. (2001). Essential Statistics.

[31] Liu H., Song B., Qin H., Qiu Z. (2013). An adaptive-ADMM algorithm with support and signal value dectection for compressed sensing. IEEE Signal Process. Lett..

[32] Gan L. Block compressed sensing of natural images.

[33] Candès E.J., Romberg J. L1-Magic: Recovery of sparse signals via convex programming. http://www.acm.caltech.edu/l1magic/downloads/l1magic.pdf.

[34] Mun S., Fowler J. DPCM for quantized block-based compressive sensing of images.

[35] Dinh K., Shim H., Jeon B. Measurement coding for compressive imaging using a structural measurement matrix.

[36] Liu H., Song B., Tian Fang, Qin H. (2014). Joint sampling rate and bit-depth optimization in compressive video sampling. IEEE Trans. Multimed..

[37] Wallace G. (1991). The JPEG Still Picture Compression Standard. J Commun. ACM.

[38] MATLAB Central File Exchange http://www.mathworks.com/matlabcentral/fileexchange/10476-jpeg-codec.

